# Accelerated magnetic resonance imaging tissue phase mapping of the rat myocardium using compressed sensing with iterative soft-thresholding

**DOI:** 10.1371/journal.pone.0218874

**Published:** 2019-07-05

**Authors:** Gary McGinley, Bård A. Bendiksen, Lili Zhang, Jan Magnus Aronsen, Einar Sjaastad Nordén, Ivar Sjaastad, Emil K. S. Espe

**Affiliations:** 1 Institute for Experimental Medical Research, Oslo University Hospital and University of Oslo, Oslo, Norway; 2 KG Jebsen Center for Cardiac Research and Center for Heart Failure Research, University of Oslo, Oslo, Norway; 3 Bjørknes University College, Oslo, Norway; Faculty of Medical Science - State University of Campinas, BRAZIL

## Abstract

**Introduction:**

Tissue Phase Mapping (TPM) MRI can accurately measure regional myocardial velocities and strain. The lengthy data acquisition, however, renders TPM prone to errors due to variations in physiological parameters, and reduces data yield and experimental throughput. The purpose of the present study is to examine the quality of functional measures (velocity and strain) obtained by highly undersampled TPM data using compressed sensing reconstruction in infarcted and non-infarcted rat hearts.

**Methods:**

Three fully sampled left-ventricular short-axis TPM slices were acquired from 5 non-infarcted rat hearts and 12 infarcted rat hearts in vivo. The datasets were used to generate retrospectively (simulated) undersampled TPM datasets, with undersampling factors of 2, 4, 8 and 16. Myocardial velocities and circumferential strain were calculated from all datasets. The error introduced from undersampling was then measured and compared to the fully sampled data in order to validate the method. Finally, prospectively undersampled data were acquired and compared to the fully sampled datasets.

**Results:**

Bland Altman analysis of the retrospectively undersampled and fully sampled data revealed narrow limits of agreement and little bias (global radial velocity: median bias = -0.01 cm/s, 95% limits of agreement = [-0.16, 0.20] cm/s, global circumferential strain: median bias = -0.01%strain, 95% limits of agreement = [-0.43, 0.51] %strain, all for 4x undersampled data at the mid-ventricular level). The prospectively undersampled TPM datasets successfully demonstrated the feasibility of method implementation.

**Conclusion:**

Through compressed sensing reconstruction, highly undersampled TPM data can be used to accurately measure the velocity and strain of the infarcted and non-infarcted rat myocardium in vivo, thereby increasing experimental throughput and simultaneously reducing error introduced by physiological variations over time.

## Introduction

Magnetic resonance imaging (MRI) offers detailed measurements of the macroscopic and microscopic structural properties of myocardial tissue in vivo. Often of great interest is the relationship between structure and function in, for example, the investigation of disease mechanisms. Methods that facilitate the measurement of motion and deformation of the myocardium are critical to understanding the regional function of the heart, particularly in the investigation of cardiac remodelling and heart failure.

Cardiac motion and deformation can be investigated in vivo using a number of techniques, such as echocardiography Tissue Doppler Imaging and a variety of MRI techniques, including myocardial tagging [1], displacement encoding using stimulated echoes (DENSE) [2], feature tracking [3] and velocity encoded tissue phase mapping (TPM) [4]. TPM, based on phase contrast MRI, can assess myocardial strain [5,6], strain rate [7] and displacement [8] throughout the cardiac cycle with high spatial and temporal resolution [9]. It is the only method able to measure the velocity directly on pixel-by-pixel basis, and is less susceptible to through-plane motion effects than for example feature tracking. However, cardiac TPM acquisition is time consuming [5], which calls for methods to reduce acquisition time. Long acquisition times are challenging in several aspects; it increases sensitivity to physiological instability, limits the achievable data yield during an examination and reduces the maximum available experimental throughput. In preclinical research, long examination times also increase the influence of anesthesia-related cardiodepressive effects. Reducing acquisition time in preclinical MRI is therefore highly desirable.

Compressed sensing (CS) allows a full image to be recovered from a subset of the complete dataset. If data can be sparsely represented in a domain, for instance by a wavelet transform, the full dataset can be represented by fewer samples than required by the Shannon-Nyquist sampling theorem [10]. CS has been used to accelerate a number of MRI protocols [11], and has previously been utilised in conjunction with SENSitivity Encoding (SENSE) to reconstruct self-gated TPM data in humans [12,13]. In the latter case, CS was utilised in order to reconstruct an undersampled self-navigated TPM dataset such that the acquisition time was comparable to previously developed prospectively ECG gated methods [14]. No studies have previously investigated the impact of CS reconstruction in TPM by directly comparing fully sampled and CS-reconstructed undersampled TPM.

In this study, we aimed to accelerate TPM by exploiting CS reconstruction. We investigated the effect of CS reconstruction of undersampled TPM data on the estimation of myocardial velocity and strain. We compared fully sampled data with CS reconstruction of both retrospectively (i.e. simulated) and prospectively undersampled datasets. Evaluation of the retrospectively undersampled data isolates the influence of undersampling and CS reconstruction on data quality, as opposed to physiology, segmentation and hardware. The prospective data demonstrates the feasibility of the method.

## Materials and methods

### Compressed sensing

The theoretical basis for CS as it applies to MRI has been well documented previously [10]. Briefly, the CS reconstruction process can be stated as a constrained optimization problem, where $l_{1}$ norm minimisation promotes sparsity, and the $l_{2}$ norm constraint promotes data consistency [11]:

$$min\{{\left\|Tx\right\|}_{1}\}\;subject\;to\;{\left\|Rx-k\right\|}_{2}\leq \varepsilon$$ [1]

Where *T* represents the complex image transform used to sparsify the complex image data, *x*. *Rx* is the partial Fourier transform of the image data, i.e. minus the k-space data that was not acquired due to undersampling. *k* is the measured k-space data, and *ε* represents the noise contained in *k*.

### Sparsifying transform

In this study, we used a pixelwise temporal Fourier transform as our sparsifying transform (*T)* exploiting the quasi-periodic nature of cardiac TPM images [10].

### $l_{1}$-norm minimisation

Minimisation of the $l_{1}$-norm was achieved by iterative soft-thresholding. Soft-thresholding is a de-noising filter that for a given input value *y* and a given threshold value λ outputs a value *x* according to the following equation:

$$x=\left\{ \begin{array}{l} y+\lambda \;\mathrm{if}\;y\;<-\lambda \\ \;0\;\mathrm{if}\;|y|<\lambda \\ y-\lambda \;\mathrm{if}\;y\;>\lambda \end{array}\right.$$ [2]

### Generation of undersampling masks

**Fig 1:**
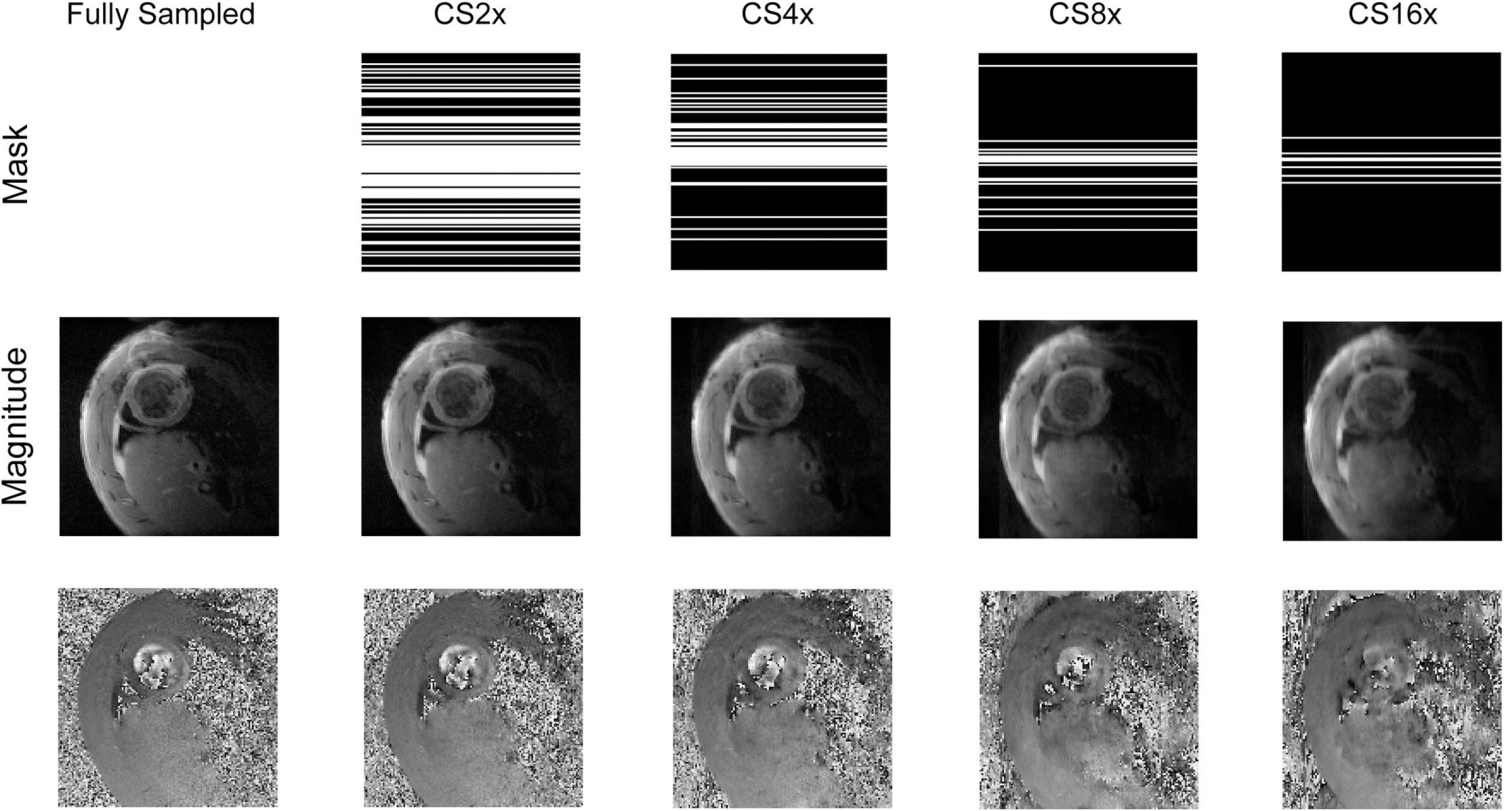
Undersampling masks. An example of representative undersampling masks for different undersampling factors, together with the magnitude data from the CS reconstructions.

In order to create undersampling masks that satisfied the incoherence property for CS for our chosen transform, a centre-weighted random number generator was used to select the phase encoded data lines to be acquired for each slice (Fig 1) [11]. The probability of acquiring lines increased towards the centre of k-space, and a quarter of all acquired lines were acquired in the centremost portion of k-space. This was done in order to preserve image contrast [15]. The incoherence of the undersampling masks was assured by measuring the sidelobe-to-peak ratio of the Fourier transform of randomly generated undersampling masks [11]. Masks which did not satisfy a minimum required incoherence were excluded. Different undersampling masks were used for each temporal frame and each velocity encoding direction, however the same set of masks were used in each rat.

### Animal preparation

LV infarcts were induced in male Wistar-Hannover rats (~300g) by left coronary artery ligation [16]. In brief, the animals were anesthetized using O_2_ and 4% isoflurane and ventilated endotracheally. Anesthesia was maintained by O_2_ and 2–3% isoflurane. Through left sided thoracotomy, the left anterior descending artery was ligated proximally, producing non-reperfused LV infarcts. As postoperative analgesia, the rats were given Buprenorphine. 2–3 rats per cage were housed in a temperature-regulated room with a 12:12 h light-dark cycle, and access to food and water *ad libitum*. Six weeks after surgery, the animals were included in this study.

All animals were cared for according to the Norwegian Animal Welfare Act. The use of animals was approved by the Norwegian Animal Research Authority (FOTS ID 3284/10102), and conformed to the Guide for the Care and Use of Laboratory Animals published by the US National Institutes of Health and the European Convention for the Protection of Vertebrate Animals used for Experimental and Other Scientific Purposes (ETS no. 123).

In accordance with the goal of reducing number of animals used in experimental research, non-accelerated MR data from the animals included here have also been reported elsewhere [5,6,17].

### Acquisition

All MRI images were acquired on a 9.4T T/210 mm/ASR horizontal bore magnet (Agilent Technologies Inc., Santa Clara, CA) with a 4 channel surface receive-only coil. Fully sampled mid-ventricular, basal, and apical short-axis TPM datasets were acquired from non-infarcted rat hearts (N = 5) and infarcted rat hearts (N = 12) using an RF-spoiled black-blood gradient echo PC-MRI sequence [5]. Additionally, TPM datasets with 4x undersampling (CS4) were acquired from one non-infarcted and one infarcted rat. The latter was reused from the prospective experiment, whereas the non-infarcted was new. The TPM datasets were acquired with the following parameters: TE = 2.3 ms; TR = 3.2 ms; field-of-view = 50x50 mm; matrix = 128x128 (for fully sampled) or 128x32 (for CS4); slice thickness = 1.5 mm; flip angle = 7°; venc = 13.9 cms^-1^ using nine-point balanced encoding [18]. The spatial resolution was chosen to provide enough extracardiac structures for the eddy current compensation [19], as well as to keep gradient duty cycle within acceptable limits. The acquisitions were ECG triggered and respiration gated, in freely breathing animals. The fully sampled acquisitions required 10–20 minutes, depending on heart rate. The CS4 acquisition required 2–4 minutes. The heart rate was kept as stable as possible by adjusting anaesthesia depth during examination, and minimising the time between acquisitions.

### CS reconstruction

CS was performed for each of the four receiver coil elements individually in order to maximise the sparsity requirement necessary for CS. The k-space matrix for each time point of the undersampled zero-filled TPM data was transformed into the image domain, before a pixel-wise 1D Fourier transform was applied along the temporal dimension. $l_{1}$norm thresholding was then performed on the real and imaginary data. After thresholding and temporal inverse Fourier transformation of each pixel was performed, the entire image data stack was transformed back to the k-space domain. Then, a data consistency operation was performed, whereby the original undersampled data was reinserted. This process was repeated iteratively until either the criterion in Eq 3 was fulfilled, or after fifty iterations.

The threshold value was chosen based on the following criteria: the threshold value was considered optimal if, for the first CS4 TPM dataset acquired from the first rat, the change of ‖*Rx*−*k*‖_2_ between two consecutive iterations was less or equal than one percent of the difference in ‖*Rx*−*k*‖_2_ between the first two iterations, after between fifteen and fifty iterations:

$$|{\left\|{Rx}_{i}-k\right\|}_{2}-{\left\|{Rx}_{i-1}-k\right\|}_{2}|\leq 0.01\cdot |{\left\|{Rx}_{1}-k\right\|}_{2}-{\left\|{Rx}_{2}-k\right\|}_{2}|\wedge 15<i<50$$ [3]

Here, *i* is the iteration number, *k* is the original undersampled dataset, R is the partial Fourier transform for a given undersampling scheme, and *x_i_* is the reconstructed image after *i* iterations. Fifteen iterations were chosen as our minimum value to protect against signal loss from aggressive denoising.

**Fig 2:**
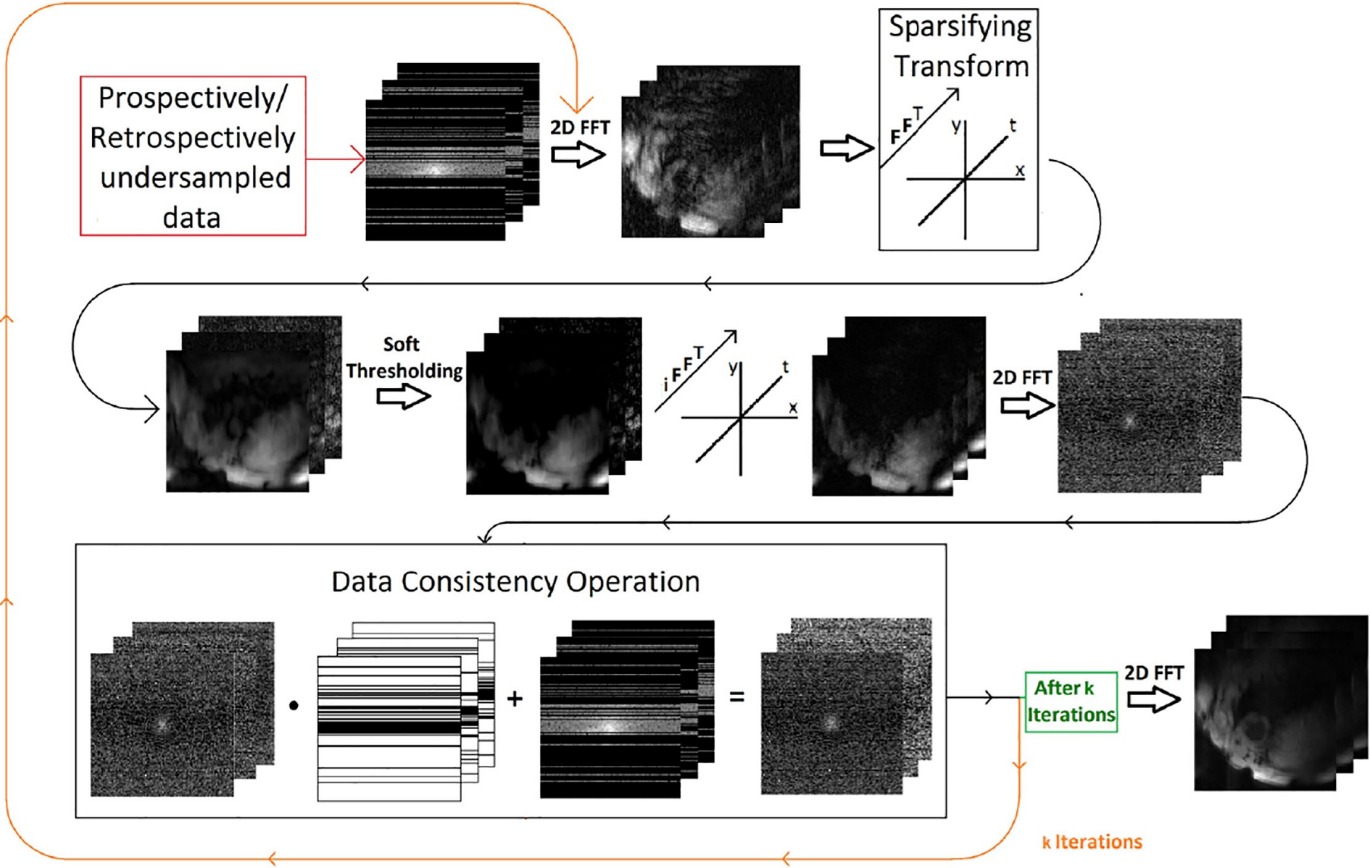
The reconstruction algorithm. An overview of the TPM CS reconstruction algorithm.

The entire reconstruction algorithm is illustrated in Fig 2.

### Retrospective CS

To isolate the effect of undersampling, we simulated undersampling using the fully sampled datasets. This was achieved by multiplying the fully sampled datasets with the different binary CS undersampling masks before performing CS reconstruction. This was done for 2x, 4x, 8x and 16x undersampling (CS2, CS4, CS8 and CS16, respectively). This allowed us to analyse the effect of undersampling alone, without the influence of physiological changes between the acquisitions.

### TPM analysis

In all reconstructed TPM images, the in-slice global radial and longitudinal velocities, and the circumferential strain of the myocardium, were calculated as previously described [5,6]. Myocardial strain offers a metric on myocardial function independent on bulk motion of the heart. We have previously shown that circumferential strain can be derived from TPM data [2]. Briefly, strain was calculated based on the displacement field of the myocardium, which was derived from temporal integration of the myocardial velocity field measured by TPM [5,20]. Regional velocities and strain were evaluated over time for 6 radial segments of the myocardium, for the purposes of Bland Altman analysis. In each region, the transmural average velocity was calculated. To isolate the error resulting from undersampling, the retrospectively undersampled CS data were analysed using the same cardiac segmentation as the fully sampled data. The prospectively undersampled CS data, however, were analysed separately.

### Intra- and interstudy variability

To evaluate intra- and interstudy variability, we performed retrospective CS on the data from midventricular slices in six animals (2 sham and 4 MI); both by repeating the acquisitions within the same session (for intrastudy variability) and repeating the examination on two consecutive days (for interstudy variability). The data was normalized to end-systole to account for slight differences in heart rate. Please note that we have reported data on intra- and interstudy variability in these animals previously [5]. Data from one of these shams are also used in the present main study.

### Statistics

All post-processing, including statistics, was performed using MATLAB 2014a (The MathWorks, Inc., USA). Reconstruction times were measured on a Lenovo Thinkstation desktop computer with an Intel Xeon CPU operating at 3.6GHz and 64GB RAM. The reconstruction algorithm was parallelised, so as to utilise the six CPU cores of the computer and speed up reconstruction. This was achieved by reconstructing data from different coil elements and with different velocity encoding separately on multiple CPU cores.

For comparing fully sampled and undersampled data, the Kolmogorov-Smirnov normality test rejected the null hypothesis for all datasets. Therefore, for comparison, we used nonparametric Bland Altman analysis. Here, we report the median and 95% percentiles of the difference as bias and limits of agreement (LOA) [21].

Global velocities and strains were compared between the sham and MI groups using the Wilcoxon rank sum test. We present the median, 25% and 75% percentiles in each group. The comparison was done between the fully sampled data and each of the four undersampling factors, and findings were compared. Since four tests were performed per parameter, a Bonferroni correction of the alpha level was used. We therefore considered p<0.05/4 = 0.0125 as statistically significant.

## Results

### Retrospective CS: Bland Altman analysis

**Fig 3:**
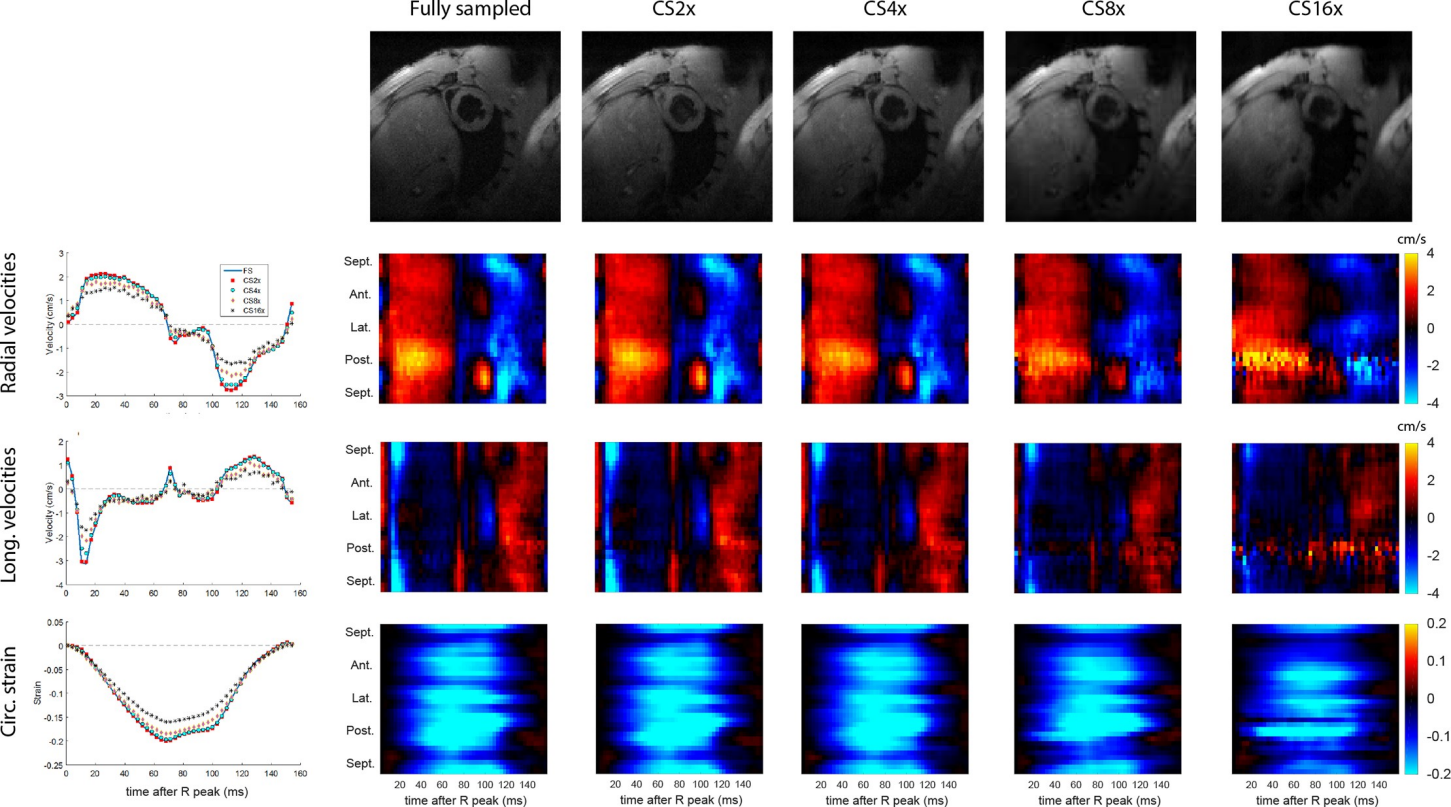
Retrospective CS results from a representative non-infarcted heart. Direct comparisons of magnitude images (1^st^ row) as well as the transmural average of global and regional radial velocities (2^nd^ row), longitudinal velocities (3^rd^ row), and global circumferential strain values (4^th^ row), for a variety of undersampling factors.

**Fig 4:**
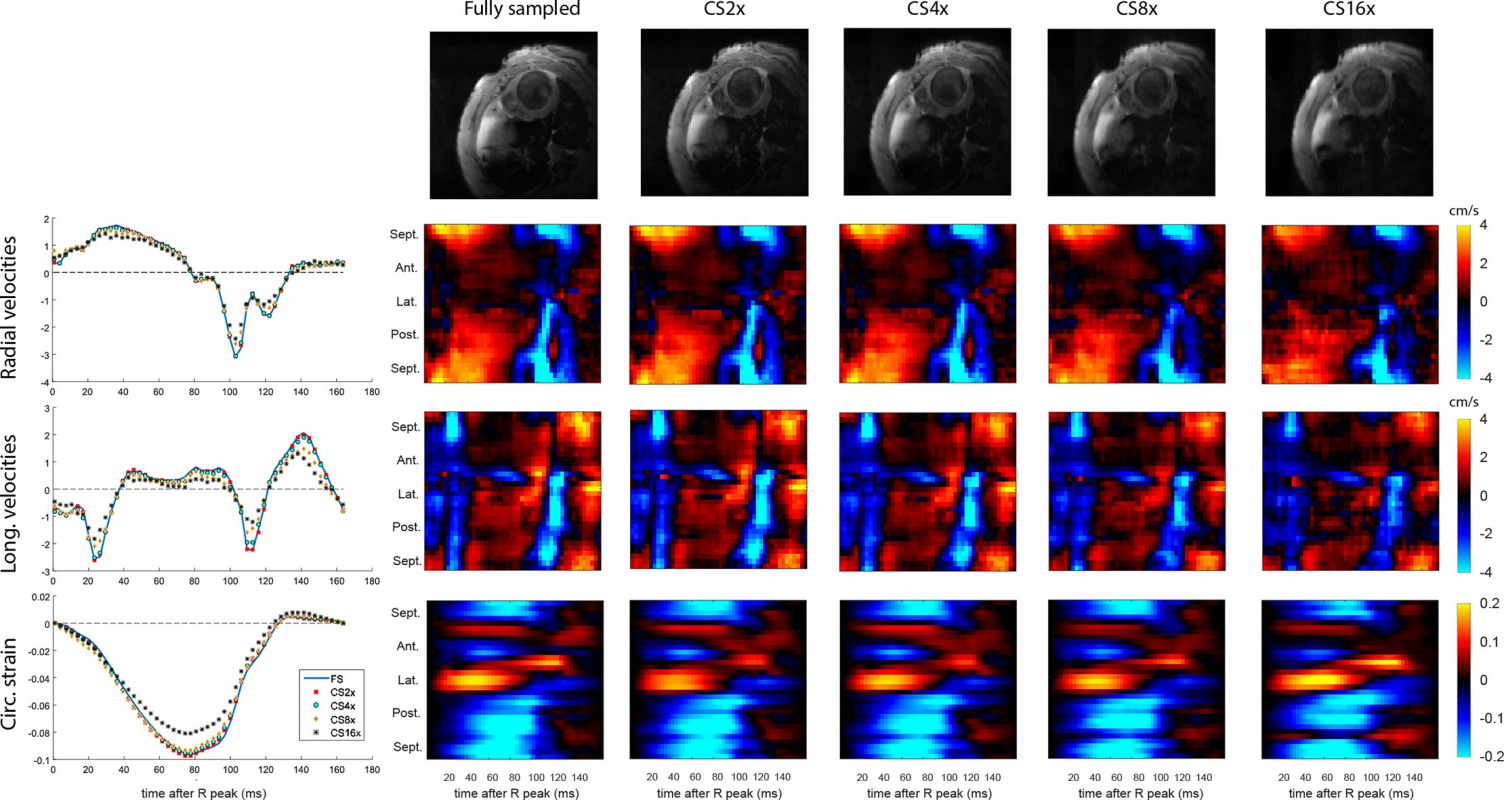
Retrospective CS results from a representative infarcted heart. Direct comparisons of magnitude images (1^st^ row) as well as the transmural average of global and regional radial velocities (2^nd^ row), longitudinal velocities (3^rd^ row), and circumferential strain values (4^th^ row) for a variety of undersampling factors.

In Figs 3 and 4, fully sampled and retrospectively undersampled datasets from mid-ventricular slices of one non-infarcted (Fig 3) and one infarcted (Fig 4) rat are compared. In the magnitude images (top rows in both figs), we can see that smoothing and signal loss increase with undersampling factor. The global velocity and strain curves show a relatively close agreement for up to 4x undersampling. By visual inspection, the regional velocities and strains are well preserved for undersampling factors up to 4x. Some artefacts are introduced in CS8 and CS16, particularly in the posterior wall.

**Fig 5:**
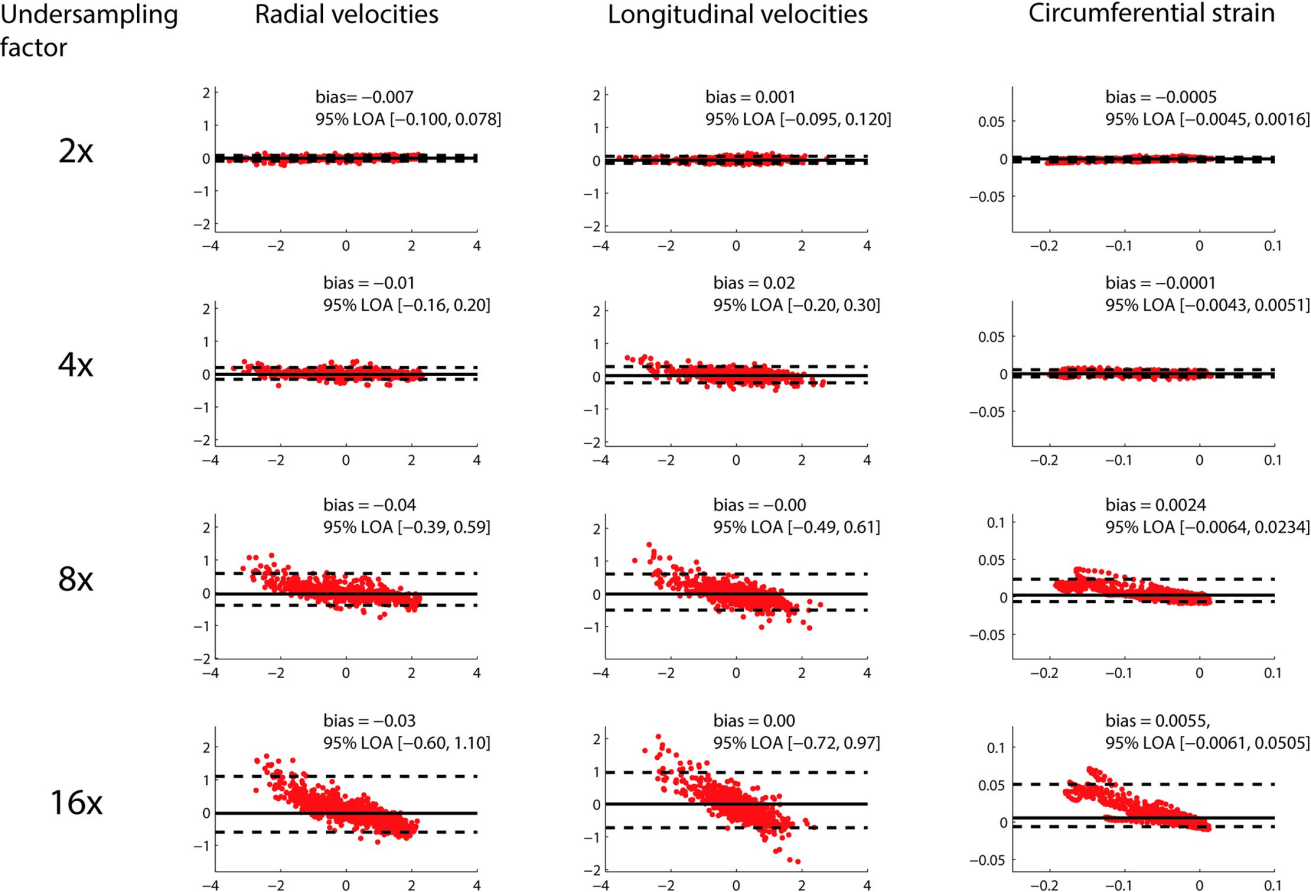
Global velocities. Bland Altman comparison of global radial velocities, longitudinal velocities, and circumferential strain from the midventricular slice, for a variety of undersampling factors.

**Fig 6:**
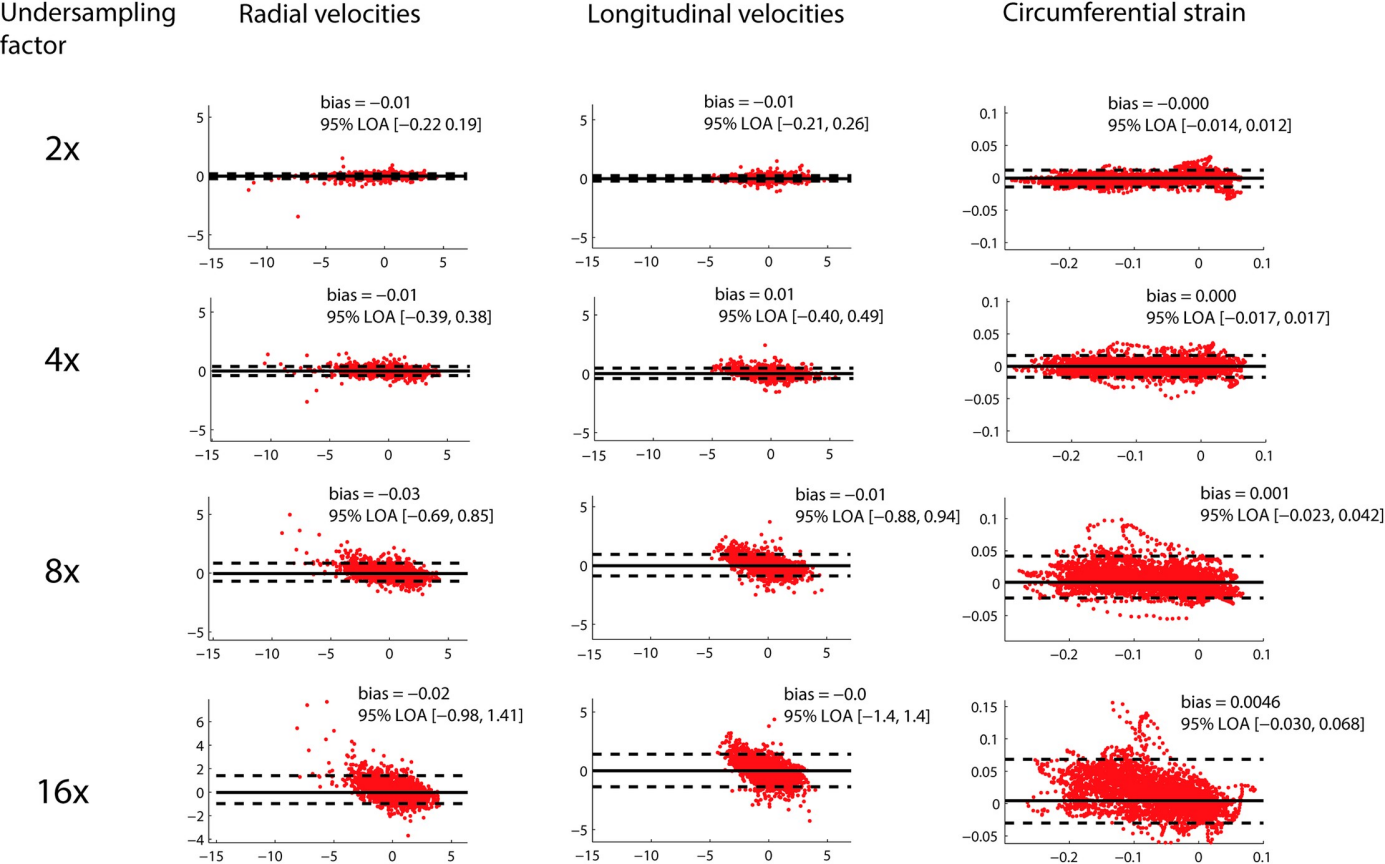
Regional velocities. Bland Altman comparison of regional radial velocities, longitudinal velocities, and circumferential strain from the midventricular slice, for a variety of undersampling factors.

Results from Bland Altman analysis of global values at the mid-ventricular level are shown in Fig 5, and for regional values in Fig 6. The width of the 95% LOA of radial and longitudinal global velocities varied from 0.18 and 0.22 cm/s for CS2 up to 1.70 and 1.68 cm/s for CS16. For regional velocities, LOAs were wider, from 0.41 and 0.47 cm/s for CS2 up to 2.4 and 2.8 cm/s for CS16. For global strain, the width of the 95% LOAs varied from 0.6% strain to 5.7% strain for CS2 up to CS16, and for regional strain the width of the LOAs varied from 2.6% strain to 9.9% strain for CS2 up to CS16.

The Bland Altman plots revealed that the circumferential strain measurements accumulated error over the duration of the cardiac cycle, which is not unexpected as the circumferential strain is calculated by integration of the velocities (Figs 5 and 6, third column).

The Bland-Altman results from the apical and basal slices are found in Table 1 (global values) and Table 2 (regional values). In the apical slice, the width of the velocity LOAs were similar to the mid-ventricular slice across all undersampling factors. For strain, the widest limit-of-agreement in the apical level was found at CS16 (global: 6.6%strain; regional: 12.9%strain).

**Table 1:** Bias and limit-of-agreements between fully sampled and undersampled data (global values).

	US	Apical		Mid-ventricular		Basal	
		Bias	95% LOA	Median	95% LOA	Bias	95% LOA
Radial velocity	2	-0.008	[-0.097, 0.076]	-0.007	[-0.100, 0.078]	0.001	[-0.108, 0.076]
(cm/s)	4	-0.01	[-0.19, 0.19]	-0.01	[-0.16, 0.20]	-0.01	[-0.16, 0.24]
	8	-0.04	[-0.44, 0.48]	-0.04	[-0.39, 0.59]	-0.02	[-0.37, 0.51]
	16	-0.04	[-0.71, 0.90]	-0.03	[-0.60, 1.10]	0.00	[-0.60, 1.02]
Long. velocity	2	0.01	[-0.11, 0.11]	0.001	[-0.095, 0.120]	-0.01	[-0.12, 0.12]
(cm/s)	4	0.02	[-0.21, 0.26]	0.02	[-0.20, 0.30]	-0.03	[-0.31, 0.25]
	8	0.01	[-0.53, 0.57]	-0.00	[-0.49, 0.61]	-0.07	[-0.80, 0.60]
	16	0.03	[-0.69, 1.03]	0.00	[-0.72, 0.97]	-0.0	[-1.2, 1.1]
Circ. strain	2	-0.03	[-0.45, 0.29]	-0.05	[-0.45, 0.16]	-0.04	[-0.42, 0.21]
(%strain)	4	-0.08	[-0.67, 1.07]	-0.01	[-0.43, 0.51]	0.02	[-0.40, 0.67]
	8	0.05	[-0.73, 3.76]	0.24	[-0.64, 2.34]	0.24	[-0.47, 1.53]
	16	0.32	[-0.81, 5.77]	0.55	[-0.61, 5.05]	0.73	[-0.70, 3.49]

Circumferential strain was calculated from temporal integration of the myocardial velocity field measured by TPM [5,20].

US = undersampling factor. LOA = limits of agreement.

**Table 2:** Bias and limit-of-agreements between fully sampled and undersampled data (regional values).

	US	Apical		Mid-ventricular		Basal	
		Bias	95% LOA	Bias	95% LOA	Bias	95% LOA
Radial velocity	2	-0.01	[-0.24, 0.20]	-0.01	[-0.22, 0.19]	0.00	[-0.22, 0.20]
(cm/s)	4	-0.01	[-0.41, 0.39]	-0.01	[-0.39, 0.38]	-0.00	[-0.36, 0.40]
	8	-0.04	[-0.74, 0.78]	-0.03	[-0.69, 0.85]	-0.02	[-0.62, 0.86]
	16	-0.0	[-1.1, 1.3]	-0.02	[-0.98, 1.41]	0.00	[-0.95, 1.47]
Long. velocity	2	0.01	[-0.24, 0.26]	-0.01	[-0.21, 0.26]	-0.01	[-0.23, 0.27]
(cm/s)	4	0.02	[-0.41, 0.50]	0.01	[-0.40, 0.49]	-0.03	[-0.48, 0.47]
	8	0.00	[-0.79, 0.99]	-0.01	[-0.88, 0.94]	-0.07	[-1.10, 0.95]
	16	0.0	[-1.2, 1.6]	-0.0	[-1.4, 1.4]	-0.0	[-1.6, 1.5]
Circ. strain	2	-0.0	[-1.5, 1.3]	-0.0	[-1.4, 1.2]	-0.0	[-1.3, 1.0]
(%strain)	4	-0.0	[-2.1, 2.2]	0.0	[-1.7, 1.7]	0.0	[-1.9, 2.2]
	8	0.0	[-2.9, 6.2]	0.1	[-2.3, 4.2]	0.2	[-2.6, 3.5]
	16	0.3	[-4.5, 8.4]	0.5	[-3.0, 6.8]	0.5	[-2.8, 7.1]

Circumferential strain was calculated from temporal integration of the myocardial velocity field measured by TPM [5,20].

US = undersampling factor. LOA = limits of agreement.

In the basal slice, the LOAs were up to 38% wider than in the midventricular slice. The widest velocity LOAs were found for longitudinal velocities at CS16; 2.3 cm/s (global) and 3.2 cm/s (regional). The widest strain LOAs were also found at CS16; 4.2%strain (global) and 10.0%strain (regional).

### Retrospective CS: Comparison of difference between groups

Next, we investigated how well any significant differences in peak velocities and time-to-peak velocities were preserved for increasing undersampling factor. The results are shown in Tables A, B, and C in S1 File. Using a Bonferroni corrected alpha level of 0.0125, all significant differences in the fully sampled data are preserved in the 2x undersampled data at all levels. In the 4x undersampled data, significance is lost for peak systolic longitudinal velocity at the basal level and peak tangential velocity at the midventricular level. In the 8x undersampled data, significance is lost also for peak systolic radial velocity at the midventricular level, as well as peak radial and longitudinal at the apical level. At 16x undersampling, significance is in addition lost for peak radial velocity in the basal level.

### Prospective CS

**Fig 7:**
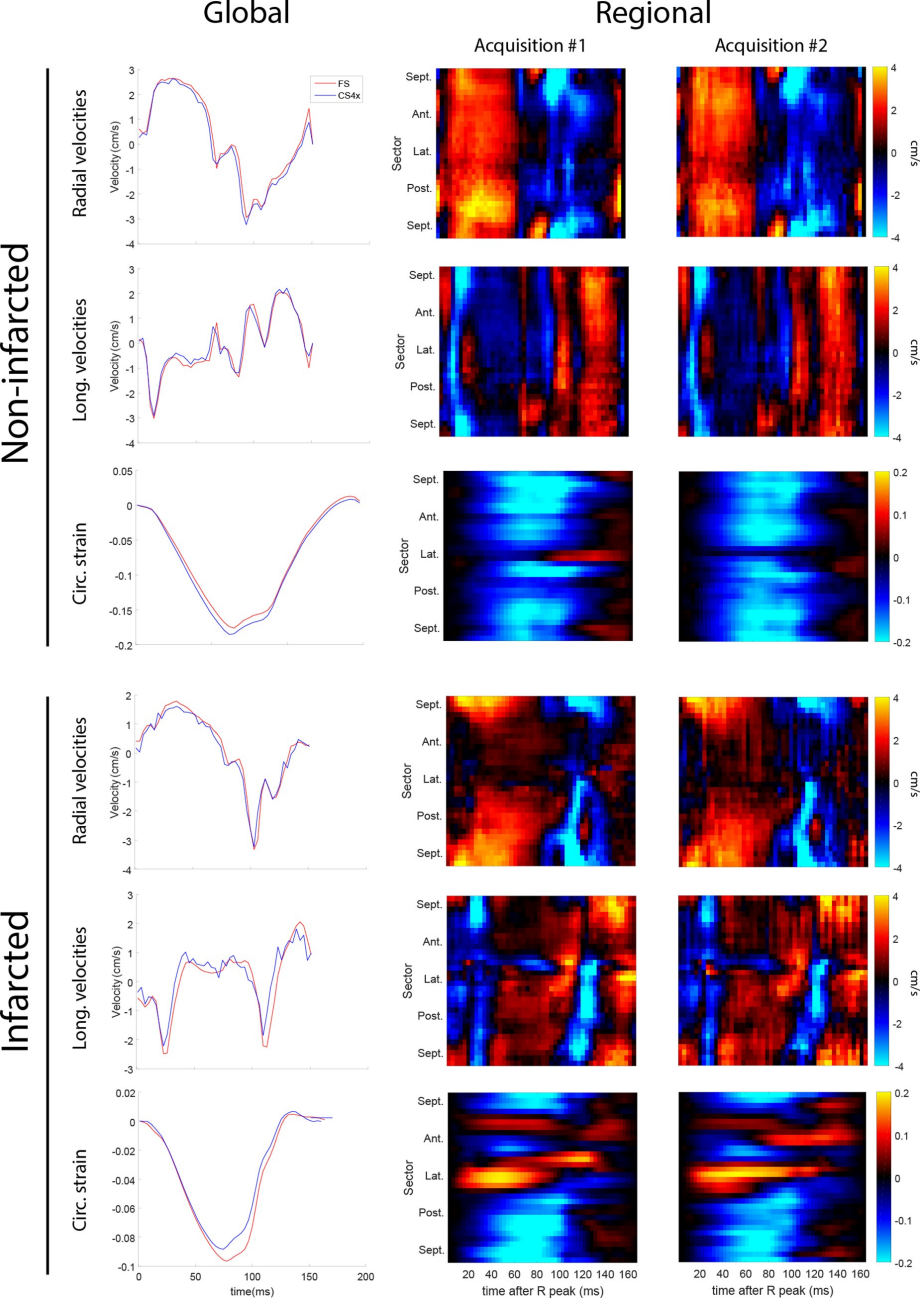
Global and regional strain. Comparison of fully sampled to 4x prospectively undersampled global and regional velocities and strain in a non-infarcted heart (top 3 rows) and an infarcted heart (bottom 3 rows).

Fig 7 show a comparison of fully sampled and CS reconstruction of *prospectively* undersampled velocities and strain from a non-infarcted and an infarcted rat heart. The global CS4 velocities and strain data agrees well with the fully sampled data in both hearts. In the regional velocities and strain, there are some visual differences, but the main features are preserved. Physiological differences could be one source of the observed discrepancies, and some of it could be caused the undersampling.

### Intra- and interstudy variability

The results of the intra- and interstudy analysis are found in Tables 3 and 4. In most datasets investigated, the width of the intra- and interstudy variability became narrower for higher undersampling factor. For intrastudy variability, the change in LOA relative fully sampled varied from 6% wider (longitudinal velocity at CS4) to 34% narrower (circumferential strain at CS16). For interstudy variability, the change in LOA relative fully sampled varied from 12% wider (circumferential strain at CS16) to 34% narrower (longitudinal velocity at CS16).

**Table 3:** Intrastudy variability.

	US	Bias	95% LOA
Radial velocity	FS	0.03	[-0.65,0.43]
	2x	0.03	[-0.60,0.46]
	4x	0.04	[-0.40,0.47]
	8x	0.05	[-0.44,0.43]
	16x	0.02	[-0.52,0.43]
Longitudinal velocity	FS	0.02	[-0.42,0.55]
	2x	0.04	[-0.40,0.60]
	4x	0.02	[-0.47,0.56]
	8x	0.00	[-0.42,0.59]
	16x	-0.01	[-0.47,0.46]
Circumferential strain	FS	-0.13	[-3.63,1.04]
	2x	-0.11	[-3.51,0.87]
	4x	-0.14	[-3.76,0.93]
	8x	-0.15	[-3.14,0.79]
	16x	-0.54	[-2.76,0.34]

The median and 95% percentile interval for the difference between two measurements on the same day. Circumferential strain was calculated from temporal integration of the myocardial velocity field measured by TPM [5,20].

US = undersampling factor. LOA = limits of agreement.

**Table 4:** Interstudy variability.

	US	Bias	95% LOA
Radial velocity	FS	0.01	[-1.09,0.78]
	2x	-0.00	[-1.16,0.75]
	4x	-0.00	[-1.04,0.72]
	8x	-0.02	[-1.01,0.62]
	16x	-0.03	[-0.81,0.66]
Longitudinal velocity	FS	-0.00	[-1.06,1.33]
	2x	0.01	[-1.03,1.50]
	4x	0.04	[-1.12,1.43]
	8x	0.05	[-0.87,1.07]
	16x	0.06	[-0.70,0.87]
Circumferential strain	FS	-0.05	[-4.08,4.13]
	2x	-0.07	[-4.29,4.41]
	4x	-0.09	[-4.37,4.54]
	8x	-0.16	[-4.23,4.96]
	16x	-0.32	[-4.23,4.12]

The median and 95% percentile interval for the difference between two measurements on two consecutive days. Circumferential strain was calculated from temporal integration of the myocardial velocity field measured by TPM [5,20].

US = undersampling factor. LOA = limits of agreement.

### Reconstruction time

CS reconstruction of a representative CS4 TPM dataset was performed both with and without parallelisation of the reconstruction algorithm. CS reconstruction took less than 5 minutes on the Lenovo desktop without parallelisation and less than two minutes with parallelisation.

## Discussion

In this study, we have shown for the first time that myocardial velocity and strain may be measured from undersampled data using CS reconstruction. This allows the scan time to be reduced, whilst preserving data fidelity. As expected, deviation from fully sampled values increases with increased undersampling factor.

### Retrospective CS data

The purpose of the retrospectively undersampled CS datasets was to assess the isolated influence of CS reconstructed undersampling on the resulting velocities and strains, as opposed to physiology, segmentation and hardware. We found that compressed sensing, particularly up to an acceleration factor of 4x, provided results in good agreement with the fully sampled data. At CS4, the width of the 95% LOAs were narrower than 0.6 cm/s for global velocities and 1.0 cm/s for regional velocities. At this level, most group-wise differences were preserved, although some significant differences were lost. At acceleration factors of 8x and 16x, edge loss and significant smoothing were evident in the images. This was also reflected in the greater deviations from the fully sampled velocity and strain measurements.

We saw somewhat increased LOAs in the strain measurement from apical and basal slices, however the increase was not dramatic. It could be a result of edge loss and the aforementioned accumulated error from integrating velocities.

We did not find that the CS reconstructed undersampled data systematically affected intra- and interstudy variability.

### Prospective CS data

Comparing prospective CS data with fully sampled data, we observed larger differences in global strains and velocities than we observed when comparing retrospective CS and fully sampled global strains and velocities. This is not unexpected since these parameters are physiology dependent, and is expected to vary somewhat over time. One notable difference in the prospective CS data for the infarcted animal is the reduced peak strain (Fig 7P). Since our retrospective data showed that CS4 do not cause a systematic underestimation of strain (Fig 6), variation in HR between acquisitions is a likely contributor.

### Implications

Lustig et al [10] introduces the concept of CS for reconstructing undersampled MRI data, focusing on exploiting sparsity in the spatial domain. In our implementation, since the TPM datasets are highly sparse as well as periodic in the temporal dimension, we chose to use a pixel-wise Fourier transform in the temporal dimension to construct our sparse domain. In general, CS-TPM differs from methods CS as applied to angiography, as the data in the latter case is far more spatially and temporally sparse, and therefore higher acceleration factors can be achieved [10].

Aside from the benefit of increased throughput, CS TPM allows us to advance our imaging capabilities in a number of ways. A single fully sampled TPM slice of the rat heart can take more than fifteen minutes to obtain without undersampling [5], rendering whole-heart TPM unfeasible without acceleration. Accelerated acquisition allows us to acquire TPM data of the whole heart during a single examination.

Strain and velocity are intrinsically three-dimensional properties, and therefore acquiring either multislice 2D or isotropic 3D TPM would provide us with a more complete picture of myocardial function.

Furthermore, CS could allow us to reduce gradient duty cycles, potentially allowing increased resolution. This could also potentially allow us to better resolve the right ventricle, as the right ventricular wall is much thinner than the left ventricular wall.

Accelerating MRI sequences, such as TPM, are also attractive since the impact of physiological variations during an examination is reduced, as well as variations introduced by anaesthesia. Additionally, sporadic changes in cardiac motion, such as cardiac rhythm abnormalities in studies of cardiac pathology, are less likely to occur during a fast acquisition than a slow one.

This method of CS is likely valuable in other phase-based CMR methods, such as DENSE.

Due to the inherent similarities between preclinical and clinical MRI, we expect no barriers in translating CS accelerated TPM to clinical usage. Future work is warranted to investigate velocity and strain derived from TPM with CS acceleration in the clinical setting. See Table B in the S1 File for theoretical breath hold times. For instance, using a fully sampled matrix size of 128x128, the theoretical breath hold time for one velocity direction at four times undersampling would be 32 heart beats, corresponding to 24 s for 80 bpm. To reduce total acquisition time, an interleaved velocity encoding approach could be considered, however at the cost of reduced temporal resolution.

### Other acceleration methods

Compared to other acceleration techniques, the proposed CS method has several advantages. Intrinsically, the undersampling factor is equal to the acceleration factor, whereas other acceleration methods might require reference acquisitions, requiring more time [22]. Parallel imaging methods also rely on the number of channels, whereas our method could readily be applied to any receiver coil configuration.

Still, TPM is well suited for parallel imaging; Jung et al have demonstrated the potential for a spatiotemporal parallel imaging technique (PEAK-GRAPPA) for phase-contrast data [22], and Simpson et al investigated acceleration of non-Cartesian TPM using SENSE [23], showing that TPM data can be acquired within a breath-hold. Since CS and parallel imaging is not mutually exclusive, further investigation into the potential benefit of combining parallel imaging acceleration with CS reconstruction in TPM is therefore warranted.

### Future perspectives

Compressed sensing is an attractive candidate for refinement using artificial intelligence. Conceivable applications is for instance 1) determining the domain in which optimal sparsity is achieved, 2) determining the optimal number of iterations to be performed balancing desired reconstruction and unwanted smoothing, and 3) an automatic determination of the λ value for soft-thresholding.

### Limitations

In this study, we have not investigated the possibility of non-Cartesian acquisition, which may offer even higher incoherence and thus more effective compressed sensing reconstruction.

We have previously shown that preclinical TPM exhibits good inter- and intrastudy repeatability [5], but in the current study we have not investigated repeatability within and between sessions. Moreover, we here only present a single centre study, thus assessment of multicentre reproducibility of the CS-TPM is warranted in future projects.

## Conclusions

We have shown that Compressed Sensing reconstruction of undersampled TPM data allows measurement of velocity and strain of both the infarcted and non-infarcted rat myocardium in vivo with high accuracy and precision. This allows us to reduce acquisition time and thereby reduce the impact of physiological variations on the data. Additionally, more data can now be acquired within a single examination, allowing us to consider advanced methods such as whole-heart TPM.

## Supporting information

S1 File Table A. Velocities and strain at the basal level, fully sampled vs 2x, 4x, 8x and 16x undersampled. Table B. Velocities and strain at the midventricular level, fully sampled vs 2x, 4x, 8x and 16x undersampled. Table C. Velocities and strain at the apical level, fully sampled vs 2x, 4x, 8x and 16x undersampled. Table D. Theoretical breath hold times.
(DOCX)
