# Supplementary material for: Accelerated magnetic resonance imaging tissue phase mapping of the rat myocardium using compressed sensing with iterative soft-thresholding
Source: PLoS One. 2019 Jul 5;14(7):e0218874. doi: 10.1371/journal.pone.0218874 (PMC6611593; doi:10.1371/journal.pone.0218874)
Supplement: S1 File — Table A. Velocities and strain at the basal level, fully sampled vs 2x, 4x, 8x and 16x undersampled. Table B. Velocities and strain at the midventricular level, fully sampled vs 2x, 4x, 8x and 16x undersampled. Table C. Velocities and strain at the apical level, fully sampled vs 2x, 4x, 8x and 16x undersampled. Table D. Theoretical breath hold times. (DOCX) [file pone.0218874.s001.docx]

## Supplementary table A: Velocities at the basal level. fully sampled vs 2x. 4x. 8x and 16x undersampled

|  |  | **Fully sampled** | | | **2x undersampled** | | | **4x undersampled** | | | **8x undersampled** | | | **16x undersampled** | | |
| --- | --- | --- | --- | --- | --- | --- | --- | --- | --- | --- | --- | --- | --- | --- | --- | --- |
|  |  | *Sham (N=5)* | *MI*  *(N=12)* | *p-value* | *Sham (N=5)* | *MI*  *(N=12)* | *p-value* | *Sham (N=5)* | *MI*  *(N=12)* | *p-value* | *Sham (N=5)* | *MI*  *(N=12)* | *p-value* | *Sham (N=5)* | *MI (N=12)* | *p-value* |
| Radial direction | Peak V_S_ | 2.45 [2.35, 2.46] | 1.92 [1.54, 2.10] | 0.0013 | 2.46 [2.35, 2.48] | 1.92 [1.57, 2.08] | 0.0013 | 2.45 [2.24, 2.48] | 1.89 [1.46, 1.98] | 0.0013 | 2.15 [1.98, 2.29] | 1.66 [1.38, 1.93] | 0.0094 | 1.87 [1.83, 2.15] | 1.50 [1.24, 1.78] | 0.0194 |
|  | TTP_S_ | 19.13 [18.34, 23.23] | 22.32 [20.77, 25.51] | 0.2809 | 19.13 [15.95, 23.23] | 22.32 [22.32, 27.11] | 0.1749 | 19.25 [18.34, 22.32] | 22.32 [20.77, 25.51] | 0.0947 | 19.13 [15.95, 20.02] | 22.32 [20.77, 25.51] | 0.0827 | 28.70 [18.43, 35.08] | 25.51 [19.13, 28.70] | 0.5472 |
|  | Peak V_D_ | 2.54 [2.28, 2.75] | 2.04 [1.71, 2.85] | 0.2343 | 2.56 [2.26, 2.77] | 2.07 [1.82, 2.97] | 0.2343 | 2.51 [2.16, 2.65] | 1.98 [1.59, 2.76] | 0.2343 | 2.36 [2.01, 2.50] | 1.83 [1.33, 2.25] | 0.0818 | 2.06 [1.57, 2.20] | 1.39 [1.01, 1.60] | 0.0268 |
|  | TTP_D_ | 114.80 [107.63, 120.14] | 105.24 [98.86, 113.21] | 0.1351 | 114.80 [106.83, 120.14] | 105.24 [102.05, 114.80] | 0.2877 | 114.80 [106.83, 120.14] | 105.48 [100.45, 114.80] | 0.2676 | 114.80 [106.83, 121.37] | 105.48 [102.05, 116.40] | 0.2873 | 117.99 [104.44, 120.14] | 105.73 [95.67, 117.99] | 0.4554 |
| Circ. direction | Peak V_S_ | 2.03 [1.77, 3.08] | 1.41 [1.03, 1.73] | 0.0194 | 2.00 [1.77, 3.06] | 1.48 [1.07, 1.72] | 0.0194 | 1.69 [1.46, 2.67] | 1.36 [1.01, 1.62] | 0.1037 | 1.27 [1.14, 2.20] | 1.25 [0.91, 1.53] | 0.3284 | 1.10 [0.94, 1.97] | 0.96 [0.64, 1.28] | 0.1946 |
|  | TTP_S_ | 3.19 [3.19, 12.78] | 20.78 [6.38, 38.27] | 0.0259 | 12.76 [3.19, 12.78] | 25.63 [9.57, 41.46] | 0.0572 | 12.76 [3.19, 12.78] | 31.96 [6.38, 41.55] | 0.074 | 12.76 [10.36, 18.40] | 30.36 [11.16, 43.15] | 0.2207 | 3.19 [3.19, 11.19] | 31.97 [11.16, 43.15] | 0.0284 |
|  | Peak V_D_ | 1.93 [1.41, 2.83] | 1.88 [1.59, 2.50] | 0.6461 | 1.87 [1.37, 2.84] | 2.09 [1.62, 2.45] | 0.9593 | 1.66 [1.17, 2.59] | 1.99 [1.53, 2.38] | 0.9593 | 1.26 [0.88, 2.05] | 1.71 [1.24, 2.05] | 0.5743 | 0.87 [0.74, 1.42] | 1.29 [0.98, 1.63] | 0.5058 |
|  | TTP_D_ | 137.13 [124.52, 154.67] | 117.99 [95.67, 129.45] | 0.0517 | 137.13 [124.52, 154.67] | 119.59 [95.67, 129.45] | 0.0514 | 137.13 [124.52, 154.67] | 119.59 [95.67, 128.16] | 0.0498 | 137.13 [124.52, 154.67] | 119.59 [97.26, 129.45] | 0.0682 | 137.13 [126.91, 154.67] | 123.07 [111.61, 131.06] | 0.0598 |
| Long. direction | Peak V_S_ | 4.31 [4.06, 4.46] | 3.32 [2.55, 3.54] | 0.0094 | 4.26 [4.00, 4.43] | 3.33 [2.55, 3.59] | 0.0094 | 3.85 [3.63, 4.07] | 3.24 [2.37, 3.54] | 0.0485 | 3.05 [2.83, 3.38] | 3.03 [1.99, 3.25] | 0.4421 | 2.58 [2.15, 2.71] | 2.52 [1.69, 2.64] | 0.7214 |
|  | TTP_S_ | 12.76 [11.96, 12.78] | 19.13 [15.95, 20.77] | 0.0003 | 12.76 [11.96, 12.78] | 19.13 [15.95, 20.77] | 0.0003 | 12.76 [11.96, 13.61] | 19.13 [15.95, 22.32] | 0.0016 | 12.76 [11.96, 13.61] | 19.13 [15.95, 22.32] | 0.0055 | 12.76 [11.96, 13.61] | 19.13 [15.95, 23.92] | 0.0019 |
|  | Peak V_D_ | 2.95 [2.62, 4.20] | 2.58 [1.97, 3.10] | 0.1946 | 2.86 [2.67, 4.19] | 2.78 [1.97, 3.10] | 0.2786 | 2.63 [2.51, 3.98] | 2.63 [1.83, 2.96] | 0.3284 | 2.26 [2.10, 3.41] | 1.96 [1.55, 2.50] | 0.16 | 1.89 [1.69, 2.91] | 1.50 [1.01, 1.88] | 0.0818 |
|  | TTP_D_ | 124.37 [106.83, 128.96] | 108.68 [95.67, 119.59] | 0.1846 | 124.37 [106.83, 127.76] | 108.68 [97.26, 119.59] | 0.2204 | 121.18 [106.83, 128.96] | 108.68 [95.67, 119.59] | 0.244 | 121.18 [106.83, 127.76] | 108.68 [97.26, 119.59] | 0.2411 | 124.37 [108.43, 125.37] | 107.09 [92.48, 121.18] | 0.2431 |

Results show median and interquartile range [25%-75% percentiles]. along with the Wilcoxon ranksum p-value.

Peak V_S_ = peak systolic velocity. Peak V_D_­ = peak diastolic velocity. TTP = time-to-peak.

Using a Bonferroni corrected alpha level of 0.0125. all significant differences in the fully sampled data are preserved in the 2x undersampled data. In the 4x. 8x and 16x undersampled data. significance is lost for peak systolic longitudinal velocity. In the 16x undersampled data. significance is lost also for peak systolic radial velocity.

## Supplementary table B: Velocities at the midventricular level. fully sampled vs 2x. 4x. 8x and 16x undersampled

|  |  | **Fully sampled** | | | **2x undersampled** | | | **4x undersampled** | | | **8x undersampled** | | | **16x undersampled** | | |
| --- | --- | --- | --- | --- | --- | --- | --- | --- | --- | --- | --- | --- | --- | --- | --- | --- |
|  |  | *Sham (N=5)* | *MI*  *(N=12)* | *p-value* | *Sham (N=5)* | *MI*  *(N=12)* | *p-value* | *Sham (N=5)* | *MI*  *(N=12)* | *p-value* | *Sham (N=5)* | *MI*  *(N=12)* | *p-value* | *Sham (N=5)* | *MI (N=12)* | *p-value* |
| Radial direction | Peak V_S_ | 2.17 [2.11, 2.35] | 1.49 [1.41, 1.76] | 0.0006 | 2.17 [2.09, 2.36] | 1.51 [1.42, 1.74] | 0.0006 | 2.06 [1.97, 2.31] | 1.44 [1.37, 1.69] | 0.0013 | 1.79 [1.75, 2.08] | 1.38 [1.28, 1.58] | 0.0023 | 1.57 [1.54, 1.94] | 1.25 [1.06, 1.45] | 0.0094 |
|  | TTP_S_ | 22.32 [21.56, 26.31] | 28.70 [25.51, 35.15] | 0.0892 | 28.70 [24.71, 28.75] | 28.70 [25.51, 35.08] | 0.5278 | 22.32 [21.56, 29.50] | 30.30 [22.32, 36.76] | 0.2411 | 28.70 [21.56, 35.08] | 28.70 [25.51, 36.76] | 0.4379 | 25.51 [16.02, 35.08] | 32.04 [28.70, 35.08] | 0.3255 |
|  | Peak V_D_ | 2.76 [2.59, 3.35] | 1.98 [1.72, 2.56] | 0.0365 | 2.84 [2.62, 3.40] | 2.10 [1.76, 2.61] | 0.0268 | 2.66 [2.42, 3.02] | 2.00 [1.67, 2.61] | 0.1037 | 2.30 [2.07, 2.51] | 1.74 [1.42, 2.15] | 0.0485 | 1.66 [1.57, 1.81] | 1.39 [1.10, 1.72] | 0.2343 |
|  | TTP_D_ | 102.69 [102.05, 110.02] | 108.43 [105.48, 113.21] | 0.3937 | 102.69 [102.05, 107.63] | 108.68 [105.24, 116.40] | 0.2094 | 105.24 [102.05, 108.92] | 108.43 [105.24, 111.61] | 0.4567 | 108.43 [102.53, 112.41] | 108.43 [107.08, 111.61] | 0.8785 | 111.61 [108.43, 115.79] | 107.08 [105.24, 114.80] | 0.4884 |
| Circ. direction | Peak V_S_ | 2.11 [1.77, 2.82] | 1.06 [0.77, 1.48] | 0.0061 | 2.07 [1.75, 2.76] | 1.05 [0.75, 1.50] | 0.0039 | 1.63 [1.54, 2.18] | 1.02 [0.72, 1.40] | 0.0136 | 1.31 [1.23, 1.59] | 0.97 [0.69, 1.14] | 0.0194 | 1.02 [0.85, 1.18] | 0.73 [0.50, 0.98] | 0.1037 |
|  | TTP_S_ | 9.57 [3.19, 10.38] | 15.95 [11.16, 46.24] | 0.0297 | 9.57 [3.19, 10.38] | 15.95 [11.16, 44.65] | 0.042 | 9.57 [3.19, 10.38] | 22.39 [9.57, 44.65] | 0.0582 | 12.76 [7.97, 13.61] | 20.80 [7.97, 59.00] | 0.3681 | 9.57 [7.97, 23.20] | 25.59 [6.38, 71.75] | 0.4632 |
|  | Peak V_D_ | 1.34 [1.30, 1.96] | 1.27 [1.16, 1.75] | 0.5058 | 1.37 [1.21, 1.92] | 1.39 [1.16, 1.74] | 0.7214 | 1.17 [1.07, 1.63] | 1.35 [1.05, 1.59] | 0.8788 | 1.02 [0.83, 1.19] | 1.18 [0.84, 1.36] | 0.4421 | 0.78 [0.69, 0.88] | 0.89 [0.77, 1.01] | 0.3284 |
|  | TTP_D_ | 143.51 [99.32, 149.09] | 119.59 [114.80, 129.15] | 0.4573 | 143.51 [99.32, 149.09] | 119.59 [114.80, 130.75] | 0.4573 | 143.51 [99.32, 149.09] | 119.59 [114.80, 129.15] | 0.4573 | 102.69 [102.05, 146.69] | 117.99 [114.80, 123.07] | 0.7757 | 98.86 [92.60, 141.91] | 116.67 [114.80, 127.86] | 0.7392 |
| Long. direction | Peak V_S_ | 3.16 [3.07, 3.46] | 2.33 [1.55, 2.66] | 0.0003 | 3.13 [2.99, 3.40] | 2.32 [1.59, 2.68] | 0.0006 | 2.70 [2.58, 2.95] | 2.22 [1.48, 2.48] | 0.0094 | 1.92 [1.89, 2.26] | 1.95 [1.20, 2.13] | 0.3827 | 1.42 [1.33, 1.77] | 1.61 [0.91, 1.84] | 0.8788 |
|  | TTP_S_ | 12.76 [9.57, 13.61] | 19.13 [15.95, 20.73] | 0.0016 | 12.76 [9.57, 13.61] | 19.13 [15.95, 20.73] | 0.0016 | 12.76 [9.57, 13.61] | 19.13 [15.95, 19.13] | 0.0016 | 12.76 [11.96, 13.61] | 19.13 [19.13, 20.77] | 0.001 | 12.76 [11.96, 13.61] | 19.13 [15.95, 22.32] | 0.0016 |
|  | Peak V_D_ | 1.92 [1.28, 2.17] | 1.45 [1.05, 1.94] | 0.3827 | 1.88 [1.30, 2.22] | 1.46 [1.13, 1.99] | 0.4421 | 1.61 [1.26, 2.09] | 1.39 [1.09, 1.87] | 0.5743 | 1.28 [1.00, 1.51] | 1.22 [0.95, 1.51] | 0.7214 | 0.84 [0.77, 1.08] | 0.89 [0.64, 1.29] | 0.799 |
|  | TTP_D_ | 130.75 [107.63, 135.35] | 121.18 [118.26, 132.34] | 0.9825 | 130.75 [107.63, 135.35] | 121.18 [116.67, 132.34] | 0.9803 | 130.75 [107.63, 135.35] | 122.78 [116.40, 132.34] | 0.9813 | 127.56 [107.63, 128.56] | 121.18 [116.40, 132.34] | 0.5727 | 124.37 [98.06, 126.17] | 130.75 [122.78, 138.72] | 0.1842 |

Results show median and interquartile range [25%-75% percentiles]. along with the Wilcoxon ranksum p-value.

Peak V_S_ = peak systolic velocity. Peak V_D_­ = peak diastolic velocity. TTP = time-to-peak.

Using a Bonferroni corrected alpha level of 0.0125. all significant differences in the fully sampled data are preserved in the 2x undersampled data. In the 4x. 8x and 16x undersampled data. significance is lost for peak systolic circumferential velocity. In the 8x and 16x undersampled data. significance is also lost for peak systolic longitudinal velocity.

## Supplementary table C: Velocities at the apical level. fully sampled vs 2x. 4x. 8x and 16x undersampled

|  |  | **Fully sampled** | | | **2x undersampled** | | | **4x undersampled** | | | **8x undersampled** | | | **16x undersampled** | | |
| --- | --- | --- | --- | --- | --- | --- | --- | --- | --- | --- | --- | --- | --- | --- | --- | --- |
|  |  | *Sham (N=5)* | *MI*  *(N=12)* | *p-value* | *Sham (N=5)* | *MI*  *(N=12)* | *p-value* | *Sham (N=5)* | *MI*  *(N=12)* | *p-value* | *Sham (N=5)* | *MI*  *(N=12)* | *p-value* | *Sham (N=5)* | *MI (N=12)* | *p-value* |
| Radial direction | Peak V_S_ | 1.87 [1.79, 1.97] | 1.24 [1.11, 1.41] | 0.0039 | 1.89 [1.79, 1.98] | 1.27 [1.07, 1.41] | 0.0061 | 1.84 [1.68, 1.94] | 1.24 [1.00, 1.41] | 0.0094 | 1.44 [1.31, 1.72] | 1.12 [0.87, 1.32] | 0.0365 | 1.16 [1.12, 1.52] | 0.90 [0.72, 1.24] | 0.0637 |
|  | TTP_S_ | 19.13 [18.34, 22.37] | 27.11 [22.32, 33.57] | 0.0407 | 19.13 [18.34, 32.84] | 27.11 [22.38, 35.16] | 0.2014 | 19.13 [18.34, 24.76] | 28.70 [22.38, 38.27] | 0.043 | 19.13 [19.13, 30.35] | 35.08 [25.51, 35.16] | 0.2437 | 19.13 [15.95, 35.13] | 31.89 [23.92, 43.05] | 0.2838 |
|  | Peak V_D_ | 2.63 [2.21, 3.63] | 1.58 [1.36, 2.18] | 0.0136 | 2.68 [2.22, 3.65] | 1.66 [1.37, 2.26] | 0.0136 | 2.43 [2.08, 3.39] | 1.52 [1.27, 2.06] | 0.0136 | 2.08 [1.58, 2.62] | 1.34 [1.02, 1.71] | 0.0637 | 1.35 [1.26, 2.10] | 0.99 [0.90, 1.25] | 0.0268 |
|  | TTP_D_ | 102.05 [101.25, 105.40] | 102.05 [95.67, 105.48] | 0.6897 | 102.05 [101.25, 105.40] | 102.05 [95.67, 105.48] | 0.5814 | 102.05 [101.25, 105.40] | 102.05 [95.67, 105.48] | 0.6959 | 102.05 [101.25, 105.40] | 102.05 [97.26, 107.09] | 0.8963 | 102.05 [102.05, 105.40] | 105.24 [97.26, 105.24] | 0.8943 |
| Circ. direction | Peak V_S_ | 2.44 [2.23, 2.96] | 0.97 [0.86, 1.37] | 0.0006 | 2.45 [2.21, 2.96] | 0.97 [0.86, 1.40] | 0.0003 | 2.25 [2.02, 2.59] | 0.95 [0.79, 1.31] | 0.0003 | 2.15 [1.73, 2.23] | 0.78 [0.67, 1.11] | 0.0003 | 1.46 [1.32, 1.78] | 0.69 [0.50, 0.84] | 0.0006 |
|  | TTP_S_ | 15.95 [11.96, 15.97] | 11.16 [3.19, 15.95] | 0.2847 | 15.95 [12.02, 15.95] | 11.16 [3.20, 15.95] | 0.2789 | 12.84 [11.96, 15.95] | 11.16 [3.20, 20.73] | 0.627 | 15.95 [15.15, 18.41] | 11.16 [3.19, 22.32] | 0.3633 | 19.13 [14.43, 26.31] | 15.95 [11.16, 27.23] | 0.6267 |
|  | Peak V_D_ | 1.78 [1.47, 2.17] | 1.37 [1.09, 1.63] | 0.1037 | 1.77 [1.47, 2.15] | 1.34 [1.11, 1.60] | 0.0818 | 1.46 [1.33, 1.91] | 1.29 [1.01, 1.50] | 0.1296 | 1.11 [0.97, 1.66] | 0.98 [0.81, 1.29] | 0.3284 | 1.00 [0.63, 1.30] | 0.79 [0.60, 0.97] | 0.4421 |
|  | TTP_D_ | 133.94 [93.40, 146.69] | 119.59 [99.08, 124.66] | 0.3675 | 133.94 [93.40, 146.69] | 115.07 [99.08, 125.97] | 0.3626 | 133.94 [95.79, 146.69] | 124.37 [106.83, 127.56] | 0.5824 | 102.05 [95.79, 144.30] | 126.26 [113.21, 127.56] | 0.8536 | 98.86 [83.83, 100.45] | 119.85 [102.05, 129.15] | 0.0229 |
| Long. direction | Peak V_S_ | 2.78 [2.73, 2.87] | 1.83 [1.47, 2.14] | 0.0013 | 2.78 [2.71, 2.85] | 1.82 [1.47, 2.09] | 0.0006 | 2.55 [2.38, 2.60] | 1.71 [1.29, 1.98] | 0.0039 | 2.03 [1.89, 2.09] | 1.34 [1.04, 1.77] | 0.0194 | 1.72 [1.63, 1.75] | 1.05 [0.76, 1.45] | 0.0039 |
|  | TTP_S_ | 12.76 [9.57, 18.40] | 19.13 [15.95, 20.73] | 0.0356 | 12.76 [9.57, 18.40] | 19.13 [15.95, 20.73] | 0.0356 | 12.84 [11.96, 32.69] | 19.13 [15.95, 20.73] | 0.4124 | 28.70 [12.82, 32.69] | 17.58 [15.95, 19.13] | 0.7731 | 31.89 [15.22, 31.89] | 17.58 [15.95, 33.48] | 0.6962 |
|  | Peak V_D_ | 1.85 [1.44, 2.52] | 1.45 [0.91, 1.72] | 0.16 | 1.85 [1.52, 2.55] | 1.47 [0.97, 1.72] | 0.1037 | 1.86 [1.40, 2.37] | 1.39 [0.83, 1.65] | 0.1037 | 1.63 [1.20, 2.03] | 1.19 [0.71, 1.43] | 0.0818 | 1.12 [0.88, 1.71] | 0.89 [0.66, 1.10] | 0.1946 |
|  | TTP_D_ | 95.67 [94.87, 110.23] | 111.88 [102.05, 119.86] | 0.3371 | 95.67 [95.67, 111.82] | 111.88 [102.05, 119.86] | 0.4308 | 95.67 [94.87, 111.82] | 111.88 [102.05, 118.27] | 0.3436 | 95.67 [94.08, 110.23] | 111.88 [102.05, 118.27] | 0.2621 | 95.67 [94.87, 115.01] | 108.94 [103.64, 117.99] | 0.4257 |

Results show median and interquartile range [25%-75% percentiles]. along with the Wilcoxon ranksum p-value.

Peak V_S_ = peak systolic velocity. Peak V_D_­ = peak diastolic velocity. TTP = time-to-peak.

Using a Bonferroni corrected alpha level of 0.0125. all significant differences in the fully sampled data are preserved in the 2x and 4x undersampled data. In the 8x and 16x undersampled data. significance is lost for peak systolic radial velocity. In the 8x undersampled data. significance is also lost for peak systolic longitudinal velocity.

## Supplementary table D: Theoretical breath hold times

| **Patient heart rate** | **50 bpm** | **60 bpm** | **70 bpm** | **80 bpm** | **90 bpm** | **100 bpm** | **110 bpm** |
| --- | --- | --- | --- | --- | --- | --- | --- |
| *Fully sampled* | 153.6 s | 128.0 s | 109.7 s | 96.0 s | 85.3 s | 76.8 s | 69.8 s |
| *2x undersampled* | 76.8 s | 64.0 s | 54.9 s | 48.0 s | 42.7 s | 38.4 s | 34.9 s |
| *4x undersampled* | 38.4 s | 32.0 s | 27.4 s | 24.0 s | 21.3 s | 19.2 s | 17.5 s |
| *8x undersampled* | 19.2 s | 16.0 s | 13.7 s | 12.0 s | 10.7 s | 9.6 s | 8.7 s |
| *16x undersampled* | 9.6 s | 8.0 s | 6.9 s | 6.0 s | 5.3 s | 4.8 s | 4.4 s |

The table show the theoretical breath hold times for one velocity direction, over a variety of heart rates and undersampling factors.
